# Electric and Manual Oral Hygiene Routines Affect Plaque Index Score Differently

**DOI:** 10.3390/ijerph182413123

**Published:** 2021-12-12

**Authors:** Shaima Bahammam, Chia-Yu Chen, Yoshiki Ishida, Akito Hayashi, Yutaka Ikeda, Hiroaki Ishii, David M. Kim, Shigemi Nagai

**Affiliations:** Department of Oral Medicine, Infection and Immunity, Harvard School of Dental Medicine, Boston, MA 02115, USA; shaima_bahammam@hsdm.harvard.edu (S.B.); yoshiki_ishida@hsdm.harvard.edu (Y.I.); akito.11@icloud.com (A.H.); yut@silk.ocn.ne.jp (Y.I.); hiroakiishii0106@me.com (H.I.); dkim@hsdm.harvard.edu (D.M.K.); shigemi_nagai@hsdm.harvard.edu (S.N.)

**Keywords:** manual toothbrushes, electric toothbrushes, oral hygiene, questionnaire, plaque index score

## Abstract

This cross-sectional study aimed to examine the oral hygiene behaviors in the general population and identify factors affecting oral hygiene behaviors and plaque removal efficacy. A survey was distributed to patients through 11 dental practices in Japan, and each patient’s plaque index score (PIS) was recorded. In total, 1184 patients participated (521 women and 660 men), with 84.04% using manual toothbrushes (MTBs) and 15.96% using electric toothbrushes (ETBs). ETB users had a significantly lower PIS compared to MTB users (*p* = 0.0017). In addition, a statistically significant difference in the PIS was detected in relation to the frequency of brushing per day (≥2 times) and time spent on brushing (≥1 min). Some MTB users spent less than 1 min brushing, while all ETB users spent at least 1 min brushing, and extended brushing periods significantly improved the PIS for the MTB users. MTB users tend to replace brush heads more frequently than ETB users, and the frequency of replacement affected the PIS significantly (*p* < 0.01) for the MTB users. The status of dental treatment (first visit, in treatment versus recall) also significantly affected the PIS (*p* < 0.01). The ETB was more effective than the MTB in terms of better plaque removal and reduced frequency of brush head replacement.

## 1. Introduction

The dental biofilm comprises a complex microbial community that develops in a highly organized sequence of events; it is a three-dimensional self-protected structure in which microcolonies are embedded in an extracellular polymeric substance [1]. If dental plaque remains undisturbed, the ecologic environment of the dental plaque favors colonization of pathogenic bacteria. This pathogenic dental plaque biofilm is the primary etiology of dental caries and periodontal diseases [1,2]. It has been found that more than twice as many adults who reported not brushing their teeth have caries compared to those who reported brushing their teeth twice a day [3]. Multiple studies have shown a positive correlation between dental plaque and gingivitis [1,4,5,6,7], and persistent gingivitis is a risk factor for the development of periodontal disease and future tooth loss [8].

Mechanical supragingival plaque removal by self-care products is the most important measure, along with frequent dental hygiene recalls for optimum oral health. Toothbrushes are the most widely used device to control supragingival plaque [9]. However, most patients do not perform adequate dental hygiene to eliminate dental plaque due to a lack of knowledge of the proper technique [10]. In addition, the practice of brushing can be time-consuming, tedious, and challenging, especially for patients with decreased manual dexterity [11]. Electric toothbrushes (ETBs) were first introduced in the American market in 1960 by the Squibb company under the name Broxodent [12]. Since then, their designs have improved with circular, elliptical, oscillating, or rotating motions to optimize plaque removal efficacy.

ETBs can be more effective than a manual toothbrush (MTB) in multiple ways. New technologies facilitated the development of interactive electric toothbrushes that have built-in time, pressure control, and real-time feedback on brushing performance through a mobile app linked with the electric toothbrush. However, they can be expensive, and not all patients can afford one, unlike MTBs. Studies have shown that interactive ETBs have allowed for increased plaque removal efficiency [13]. An MTB often requires a more careful brushing technique to be followed as recommended by the American Dental Association (ADA) [14], while ETBs can be easier to use and are more attractive, especially to children. ETBs have been proven to be more effective in improving plaque removal, especially in patients with inadequate oral hygiene, such as in children [15], adolescents wearing fixed orthodontic appliances [14], and individuals with mental disabilities [16].

Multiple studies and systematic reviews where different forms of electric and manual toothbrushes were studied showed ETBs to be generally more effective in removing plaque than manual toothbrushes in short- and long-term clinical trials [17,18,19,20]. In this cross-sectional study, we aimed to examine the oral hygiene behaviors in the general population and identify factors affecting oral hygiene behaviors. We correlated the efficacy of plaque removal with different oral hygiene routines defined by the type of toothbrush used (ETB vs. MTB), duration and frequency of brushing, and brush head/toothbrush replacement. Secondary analysis was performed for ETBs and MTBs to investigate whether ETBs could reduce time spent on brushing.

## 2. Materials and Methods

### 2.1. Participants

This cross-sectional study was approved by the Institutional Review Board of Harvard Medical School (IRB20-1284). All participants were given sufficient explanation about the study and signed consent forms. Participants were recruited from 11 dental offices in Japan from April to October 2020. All patients above the age of 18 years who visited one of these dental offices and appeared to have normal mental and physical abilities were asked to participate in the study. Patients who agreed to participate were registered in the study.

### 2.2. Survey

**Figure 1:**
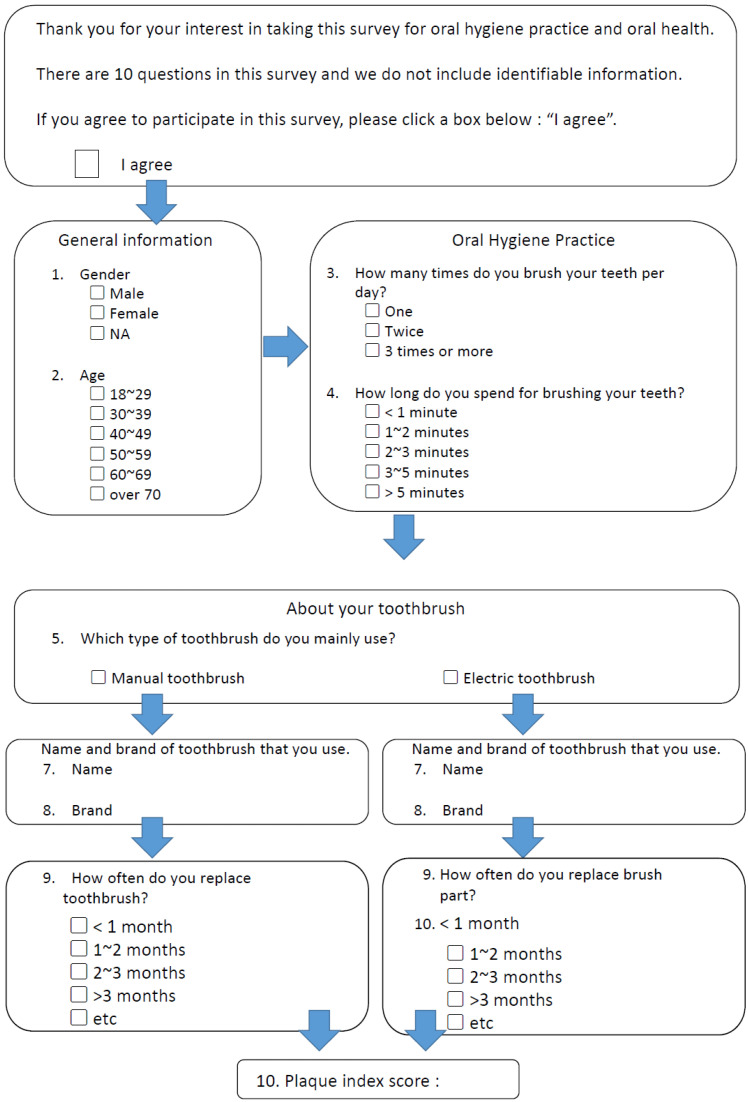
Survey administered to the participants.

All participants completed a survey consisting of their demographic characteristics (age and sex) and oral health behavior, including the type of toothbrushes used (manual or electric), frequency of brushing per day, time spent on brushing, and frequency of toothbrush/brush head replacement (Figure 1). We designed the survey questions based on published studies [21,22,23], which were pilot-tested by comparable groups of adults to evaluate their effectiveness.

### 2.3. Status of Dental Treatment

The status of the participants’ dental treatment was recorded. They were either new patients to the dental practice or established patients undergoing active treatments or coming for routine recall visits.

### 2.4. Plaque Index Score (PIS)

An oral examination, including the use of a plaque-disclosing agent (GUM^®^ RED-COTE^®^ Tablets, Sunstar, Osaka, Japan), was performed for all participants. The plaque index score (PIS), based on the 1972 O’Leary Index, was measured for each participant [24]. Each tooth was divided into 4 sites (buccal, lingual, mesial, and distal), and if plaque was present, it was recorded on the corresponding tooth site. Then, the PI was calculated as a percentage using the following formula: $\frac{Number\;of\;sites\;with\;plaque}{Total\;number\;of\;sites\;evaluated}\times 100$. Prior to the initiation of the study, participating dentists and hygienists were calibrated to ensure that the use of the disclosing agent and the subsequent recording of the presence of plaque were standardized.

### 2.5. Statistical Analysis

A sample size calculation set at a 5% level of significance and based on a 10% use of electric toothbrushes among Japanese people was performed [25]. The minimum number of respondents per group was 138. One-way ANOVA, post hoc Tukey’s HSD test, and the Welch’s *t*-test were used to analyze the association between the PIS and oral hygiene behaviors. Differences in the PIS between participants using manual and those using electric toothbrushes were analyzed using an independent *t*-test. The association among sex, age, and hygiene behaviors was analyzed using a chi-square test. The level of significance was set at α = 0.05 (two-tailed).

## 3. Results

**Figure 2:**
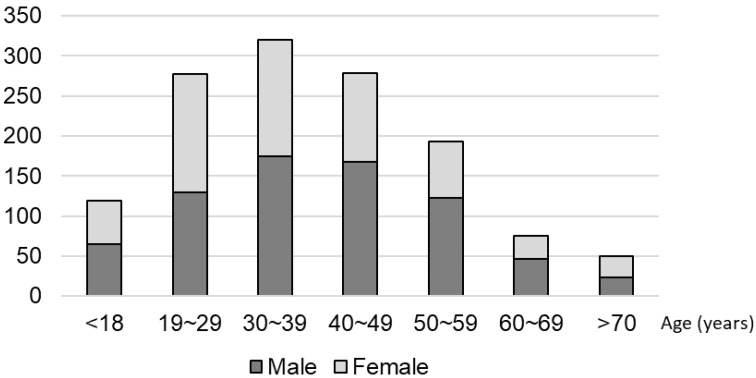
Demographic distribution of the participants.

In total, 1184 patients (660 men and 521 women) participated from 11 dental practices. The majority of these participants were in the 20–50 years age groups (Figure 2).

### 3.1. Oral Hygiene Routines for All Participants

Of the total participants, 995 (84.04%) used manual toothbrushes (MTBs) and 189 (15.96%) used electric toothbrushes (ETBs). There were five main brands of MTBs and four main brands of ETBs used among the participants, with Lion being the most commonly used by MTB users (19.4%), and Philips Sonicare being the most widely used among ETB users (43%). When it came to brushing frequency and the time spent on brushing per day, most patients brushed twice a day and spent between 2 and 5 min brushing. In terms of the frequency of toothbrush replacement, we found that most people replaced their brushes once every 1–2 months (Figure 2).

### 3.2. Factors Affecting Overall Oral Hygiene Behaviors

**Figure 3:**
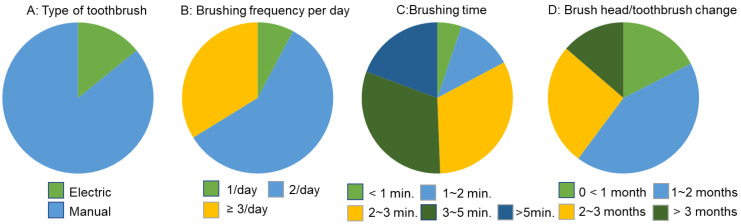
Oral hygiene routines for all participants.

Both sex and age were associated with differences in oral hygiene behaviors. Women were more likely to use ETBs (*p* < 0.00001), brushed more frequently, and replaced their brushes more regularly (*p* < 0.01). The 18–29-year-old age group represented the lowest percentage of ETB users (8%, *p* < 0.00001), older adults tended to brush their teeth more frequently (*p* < 0.01), spent more time brushing (*p* < 0.001), and replaced their brushes more often (*p* < 0.01) (Figure 3).

### 3.3. Association among PIS, Oral Hygiene Behaviors, and Status of Dental Treatment

Women had a significantly lower PIS compared to men (*p* = 0.0142). Similar PISs were recorded in all age groups; however, a significantly higher PIS was observed in participants in their twenties (46.4% ± 23.1%) compared to those in their fifties (39.0% ± 20.5) (*p* < 0.01). In addition, the PIS became significantly lower as the frequency of tooth brushing increased per day (once vs. twice *p* < 0.01; once vs. three times *p* < 0.05). Brushing for at least 2 min was important for plaque control as these participants had significantly lower PISs than those brushing less than 2 min (*p* < 0.01). The highest PIS observed was in participants who brushed for less than 1 min, and the lowest was seen in those who reported brushing for more than 5 min. The frequency of replacing brush heads/toothbrushes also influenced plaque removal efficacy. Using the same toothbrush for more than three months was associated with a significantly higher plaque score (*p* = 0.0005). Furthermore, we found that ETB users had substantially better plaque control than MTB users (*p* = 0.0004) (Table 1).

**Figure 4:**
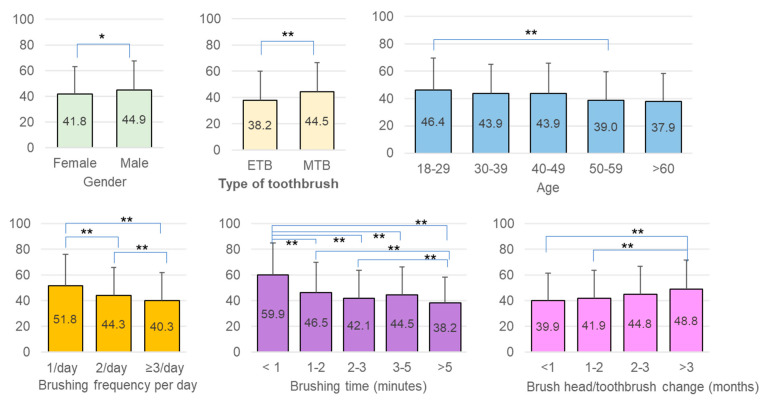
Comparison of the PIS among sex, age, and oral hygiene behaviors (* *p* < 0.05; ** *p* < 0.01).

The status of dental treatment was another factor that influenced the PIS. Participants undergoing active treatment or in routine recall with regular appointments demonstrated better oral hygiene (*p* < 0.01) (Figure 4).

### 3.4. Differences between the Manual (MTB) and Electric Toothbrush (ETB)

**Figure 5:**
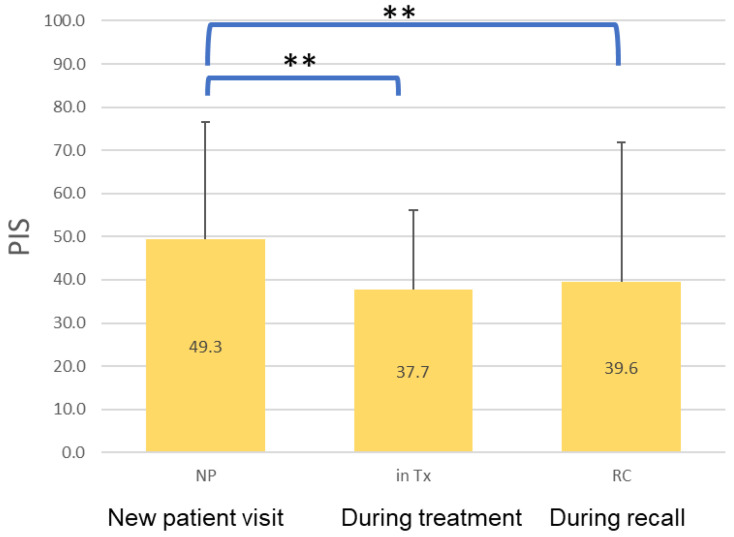
Comparison of the PIS in 3 treatment statuses (** *p* < 0.01).

In general, ETB users tended to brush more frequently and spent at least 1 min brushing. MTB users replaced their brushes more frequently, with 61.19% replacing them at least every two months, while 50% of ETB users replaced them every three months. There was no significant difference in the PIS within the ETB users regardless of the frequency of brushing, the time spent on brushing, and the frequency of brush head replacement. By comparison, brushing only once per day (*p* < 0.0001) and spending less than 1 min brushing (*p* < 0.01) were associated with a significantly higher PIS for MTB users (Figure 5).

**Figure 6:**
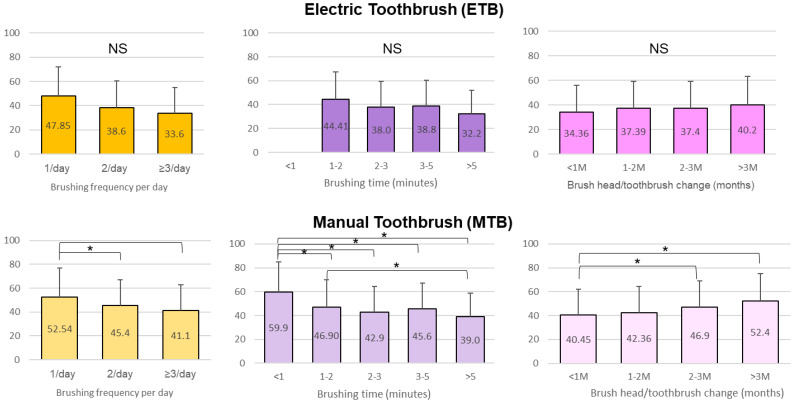
Comparison of the plaque index score (PIS) among oral hygiene routines in electric toothbrush group and manual toothbrush group (* *p* < 0.05).

**Figure 7:**
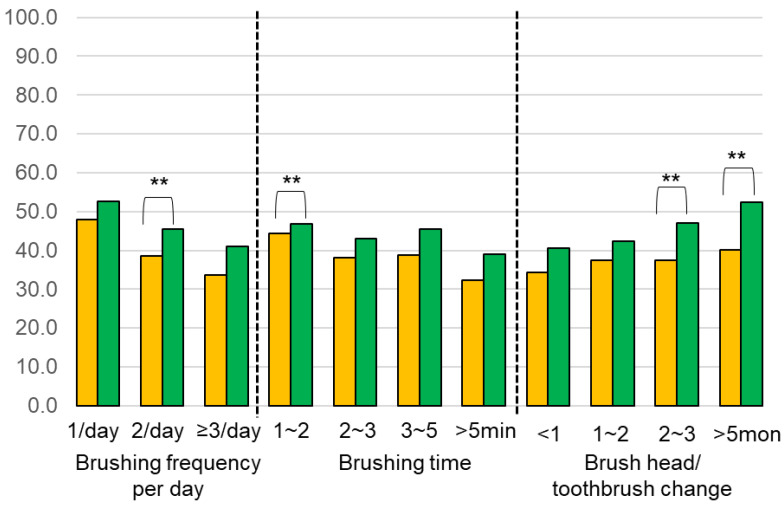
Comparison of the PIS between electric (yellow) and manual (green) toothbrushes on different oral hygiene practice behaviors (** *p* < 0.01).

Comparing MTB and ETB users, we found that brushing twice a day with an ETB was more effective for plaque removal (*p* = 0.0015) (Figure 6 and Figure 7). When spending only 1~2 min brushing, ETB users had a significantly lower PIS compared to MTB users (*p* = 0.0110) (Figure 6 and Figure 7). In addition, the ETB required less frequent brush head replacement and continued to show better plaque removal efficacy even up to 5 months (*p* = 0.0022) (Figure 6 and Figure 7).

## 4. Discussion

This cross-sectional study aimed to examine the association between plaque index scores and oral hygiene routines and further investigate the differences between manual and electric toothbrushes. Current guidelines for dental hygiene practices are based on manual toothbrushes [26]. However, there are so many electric toothbrushes available on the market, and dentists/hygienists always find themselves being asked by patients whether electric toothbrushes are more effective than manual toothbrushes and how to use them.

In this study, after examining 1184 participants for their PIS, we found that ETB use was associated with a significantly lower PIS compared to MTB use. This is consistent with multiple studies reported in a Cochrane review in 2014 [20]. All forms of electric and manual toothbrushes were included and showed that electric toothbrushes are more effective in removing plaque than manual toothbrushes in both the short and long term. Other clinical trials support this finding; however, they often recruited a limited number of participants, generally with good oral hygiene, and trained them with the proper brushing technique, had them brush in the dental office for a specified duration while being supervised by the dental staff, which probably made the subjects more conscious of their brushing technique, and that could have affected the outcome [27,28,29,30,31,32,33]. With the present investigation being a cross-sectional study, we captured a large population in a natural state that was more representative of the actual oral hygiene behavior among the public. In addition, other factors that could affect the efficacy of brushing are being investigated. To the best of our knowledge, this is the first observational study that reported the association between the PIS and a patient-reported oral hygiene practice routine.

We found that MTB users tended to replace brush heads more frequently than ETB users. This could be explained by the fact that an MTB is more likely to splay after being used for three months compared to an ETB [34]. In this study, we also observed that the frequency of replacement significantly affected the MTB users’ PIS. This could also be attributed to the fact that manual brushes tend to wear faster, and toothbrush wear was significantly associated with higher plaque scores [35]. It is essential to regularly replace a brush head, whether electric or manual, with significant wear as plaque removal efficiency will be reduced [35].

We wanted to investigate if using ETBs reduced the required time spent on brushing. We found that all ETB users spent at least 1 min brushing, and that longer brushing times did not significantly improve the PIS. By comparison, longer brushing time significantly improved the PIS for MTB users. When spending between 1 and 2 min brushing, ETBs were significantly more efficient for plaque removal compared to MTBs. In general, it has been shown in multiple experimental studies that the efficacy of tooth brushing is improved when the brushing time is increased for both manual and electric brushes [36,37]. Van der Weijden et al. tested the effectiveness of four different toothbrushes (three different electric toothbrushes compared to one manual toothbrush) in removing plaque in relation to time [36]. They found that for all brushes (electric and manual), the greater part of the efficacy is reached after 30 s of brushing per quadrant, which is 2 min for the whole mouth. The results of our study also corroborated this finding and showed that brushing at least twice a day and spending at least 2 min brushing should be recommended for MTB users. This is in agreement with a more recent systematic review that showed an increased efficacy of MTBs after brushing for 2 min [38]. Another study by Williams et al. in 2004 compared the plaque removal efficacy of a manual toothbrush to an electric toothbrush [35]. They found that both toothbrushes had statistically significantly greater plaque removal scores after 3 min than after 1 min of brushing. In our study, we only observed an improvement in the PIS with longer brushing times with an MTB.

It is possible that an MTB can be as effective as an ETB if the proper technique and a sufficient brushing duration are followed. Some clinical trials have shown that there is no difference in plaque removal between MTBs and ETBs when proper techniques are followed. In one study, subjects had their teeth brushed by a periodontist [39] and, in another study, trained dental students were the study subjects [37]. However, brushing performed by dental professionals is not representative of the brushing practice performed by patients.

In this study, an overview of the effectiveness of MTBs and ETBs was assessed based on the patient-reported variables in the duration and frequency of brushing and the frequency of toothbrush replacement. The patient self-reported response has its inherent inaccuracy, and the only objective parameter assessed was the PIS. There was also a wide variety of toothbrushes used by participants in this study. Multiple studies have shown a difference in efficacy even among different electric toothbrushes [32,36,40,41,42,43]. Future studies should incorporate more clinical parameters such as bleeding score; decayed, missing, filled (DMF) indexes; and the frequency of professional hygiene appointments per year. A retrospective investigation of the incurred dental care cost could also help in making public health recommendations.

## 5. Conclusions

Within the limitation of this cross-sectional study, we found that a higher frequency of brushing, a longer time spent on brushing (at least 2 min), and regular dental visits were associated with better plaque control. Furthermore, electric toothbrushes are more effective than manual ones in plaque removal and in reducing the frequency of brush head replacement.

## Figures and Tables

**Table 1:** Factors associated with oral hygiene behaviors. The chi-square statistics were performed to examine the relationships between background variables (sex and age) and oral hygiene behaviors.

		Type of Toothbrush	Frequency/Day	Brushing Time	Brush Head/Toothbrush Change
Gender	The chi-square	26.6906	75.7634	1.4543	14.6999
	*p* value	<0.00001	<0.00001	Not significant	<0.01
Age	The chi-square	22.7229	20.9302	79.1158	26.373
	*p* value	<0.001	<0.01	<0.001	<0.01
